# Identidication of novel biomarkers in non-small cell lung cancer using machine learning

**DOI:** 10.1038/s41598-022-21050-5

**Published:** 2022-10-06

**Authors:** Fangwei Wang, Qisheng Su, Chaoqian Li

**Affiliations:** 1 Department of Respiratory Medicine, The First Affiliated Hospital of Guangxi Medical University, Nanning, 530021 Guangxi China; 2 Department of Clinical Laboratory, The First Affiliated Hospital of Guangxi Medical University, Nanning, 530021 Guangxi China

**Keywords:** Bioinformatics, Biomarkers, Oncology

## Abstract

Lung cancer is one of the leading causes of cancer-related deaths worldwide, and non-small cell lung cancer (NSCLC) accounts for a large proportion of lung cancer cases, with few diagnostic and therapeutic targets currently available for NSCLC. This study aimed to identify specific biomarkers for NSCLC. We obtained three gene-expression profiles from the Gene Expression Omnibus database (GSE18842, GSE21933, and GSE32863) and screened for differentially expressed genes (DEGs) between NSCLC and normal lung tissue. Enrichment analyses were performed using Gene Ontology, Disease Ontology, and the Kyoto Encyclopedia of Genes and Genomes. Machine learning methods were used to identify the optimal diagnostic biomarkers for NSCLC using least absolute shrinkage and selection operator logistic regression, and support vector machine recursive feature elimination. CIBERSORT was used to assess immune cell infiltration in NSCLC and the correlation between biomarkers and immune cells. Finally, using western blot, small interfering RNA, Cholecystokinin-8, and transwell assays, the biological functions of biomarkers with high predictive value were validated. A total of 371 DEGs (165 up-regulated genes and 206 down-regulated genes) were identified, and enrichment analysis revealed that these DEGs might be linked to the development and progression of NSCLC. *ABCA8, ADAMTS8, ASPA, CEP55, FHL1, PYCR1, RAMP3*, and *TPX2* genes were identified as novel diagnostic biomarkers for NSCLC. Monocytes were the most visible activated immune cells in NSCLC. The knockdown of the *TPX2* gene, a biomarker with a high predictive value, inhibited A549 cell proliferation and migration. This study identified eight potential diagnostic biomarkers for NSCLC. Further, the *TPX2* gene may be a therapeutic target for NSCLC.

## Introduction

Non-small cell lung cancer (NSCLC), a subtype of lung cancer, is one of the most prevalent malignancies worldwide. According to studies, the prognosis for NSCLC is highly dependent on the stage of disease progression, and the earlier the disease is detected, the better the chances of survival within 5 years^1^. Patients with early-stage lung cancer often have no obvious symptoms and thus miss the best time for treatment. Moreover, metastasis is the most devastating feature of the tumor, ultimately leading to a high mortality rate^2^. Although some advances in lung cancer treatment and pharmaceutical research have been made, serious complications such as anemia and neutropenia persist, and recurrence rates and mortality in NSCLC patients are still not effectively controlled^3^. Therefore, there is an urgent need to identify reliable biomarkers in the diagnosis and prognosis of NSCLC.

With the significant advancement of microarray and sequencing technology in recent years, gene characterization based on messenger RNA expression levels has shown great promise in diagnosing cancer. For instance, the breast cancer susceptibility protein-1 (*BRCA1*) gene has been considered a predictor in breast cancer risk models. It is used as a clinical genetic test standard, while the tumor germination (*Bd*) gene is also considered a prognostic biomarker and has an independent prognostic value for disease-free survival (DFS) and overall survival (OS) in colon cancer^4^.

It has previously been reported that bioinformatics methods are used to analyze comprehensive gene expression data to obtain cancer-related biomarkers for effective prevention, diagnosis, and treatment of cancer. Machine learning (ML) methods are a branch of bioinformatics applied to various aspects of cancer research and are a hot topic in lung cancer research. For example, ML methods successfully developed and validated a predictive model for cancer-related deep vein thrombosis^5^. Lai et al. used ML methods to create gene signatures that accurately predicted prostate cancer prognosis^6^. Zheng et al. developed an integrated radiomic model using the ML method to predict the prognosis of prostate cancer patients^7^. ML methods have also been used to screen for potential biomarkers of prostate cancer and osteosarcoma^8,9^. Support vector machines (SVM), a linear classifier with maximum intervals defined in the feature space, have gradually been applied to machine learning of cancer using its efficient two-class model to classify cancer with high accuracy from a large number of genomes and efficiently extract key genes to achieve high-accuracy, low-error sample classification. In recent years, many researchers have used SVM methods to study cancer and have made significant advances. For example, Luo et al. used SVM methods to construct predictive models for synchronous lung metastases (SLM) in osteosarcoma^10^. Su et al. identified eight genes associated with colon cancer prognosis using the SVM method^11^. Cai et al. used SVM methods to construct radiomics-based models for diagnosing lung adenocarcinoma (LUAD)^12^. SVM methods also differentiated between epithelial ovarian cancer (EOC) and surrounding tissues, aiding EOC diagnosis^13^. In addition, the SVM methods successfully screened colorectal biomarkers for personalized treatment of patients with post-operative liver metastases^14^. However, there have been few studies on potential NSCLC biomarkers.

In this study, we obtained differentially expressed genes (DEGs) for NSCLC from the Gene Expression Omnibus (GEO) database. We used functional enrichment analysis to identify NSCLC-related biomarkers using an ML approach, followed by infiltrative immune cell analysis and in vitro validation of the biomarkers’ functions. Our findings may be useful in the early diagnosis of NSCLC and the mechanistic study of NSCLC.

## Materials and methods

### Data processing and DEGs screening

We obtained three microarray datasets (GSE18842, GSE32863, and GSE21933) from the Gene Expression Omnibus (GEO) public databases, and the organism parameter was set to “Homo sapiens”. The raw data from these datasets were processed using R language statistical software (version 4.1.2)^15^. Using the R ‘Limma’ package^16^, we identified DEGs between normal lung and NSCLC tissues, with an adjusted *P*-value < 0.05 and |logFC|≥ 2 as statistical significance. Heatmaps and volcano plots were plotted to show the differential expression of DEGs.

### Functional and pathways enrichment analysis

Gene Ontology (GO), Kyoto Encyclopedia of Genes and Genomes (KEGG)^17–19^, and Disease Ontology (DO) enrichment analyses of DEGs were performed using the R ‘clusterProfiler’ package^20^ and visualized using the R ‘ggplot2’ package^21^ with GO functional annotation, including biological process, cellular component, and molecular function terms. The R ‘clusterProfiler’ and R ‘org.Hs.eg.db’ packages^22^ were used to enrich DEGs using gene set enrichment analysis (GSEA). A result with an adjusted *P*-value < 0.05 and a false discovery rate < 0.05 was considered statistically different.

### ML methods to identify NSCLC biomarkers

For biomarker screening, two ML methods were used: least absolute shrinkage and selection operator (LASSO) logistic regression^23^ and support vector machine-recursive feature elimination (SVM-RFE)^24^. The algorithm LASSO used the R ‘glmnet’ package^25^, while the SVM-RFE algorithm used the R ‘e1071’ package^26^. The following are the model settings: LASSOcvfit = cv.glmnet (x,y,family = ‘binomial,” alpha = 1, type.measure = ‘deviance,’ nfolds = 10). SVM = rfeControl (functions = caretFuncs, method = “cv,” methods = “svmRadial”). The point with the lowest cross-validation in the vertical axis corresponds to the biomarker genes to be found; a difference of *P* < 0.05 was considered statistically significant.

### Validation of biomarkers

The R ‘ggpubr’ package^27^ was used to examine the biomarker expression in GSE32864. In addition, the biomarkers’ diagnostic efficiency was validated using receiver operating characteristic (ROC) curves generated with the R ‘pROC’ package^28^; *P* < 0.05 was considered statistically significant.

### Assessment and correlation analysis of infiltrating immune cells

The CIBERSORT algorithm^29^ was used to analyze the relationship between infiltrating immune cells and biomarkers; a correlation heatmap was produced using the R ‘corrplot’ package^30^ to detect the association of each immune cell with the other cells in the LUAD sample; the violin map using the R “ggplot2” package showed differences in the expression of 22 immune cells. A Spearman correlation analysis was performed between diagnostic biomarkers and infiltrating immune cells using the R “ggstatsplot” package.

### Survival analysis

The GEIPA online website (http://gepia.cancer-pku.cn/) is a dataset based on The Cancer Genome Atlas and Genotype-Tissue Expression that provides a fast and customizable web-based tool. A summary of the process is as follows: click on “survival plots” and enter the gene name, then select “LUAD,” “OS,” and “RFS,” and finally observe the *P*-value and output the graph; *P* < 0.05 was considered statistically significant.

### Cell culture and cell transfection

A549 cells were purchased from the ATCC (Shanghai, China). A549 cells were cultured in Dulbecco’s modified Eagle medium (Thermo Fisher Scientific, USA) containing 10% serum (Thermo Fisher Scientific, Wilmington, DE, USA). For si-RNA transfection, A549 cells were transfected with si-TPX2 using the Lipofectamine 2000 transfection reagent (Invitrogen, Waltham, MA, USA) according to the manufacturer’s instructions. To target TPX2, the following si-RNA sequences were used: AGCCTCAGAAGATCTCTTAG (si-TPX2).

### Western blotting

The total protein concentration extracted with lysis buffer containing protease inhibitors was measured using the bicinchoninic acid (BCA) protein assay kit (Beyotime Biotechnology Inc., Shanghai, China), and proteins were separated using a polyvinylidene fluoride membrane (Millipore, Billerica, MA, USA). Proteins were separated on 10% skim sodium dodecyl sulfate–polyacrylamide gel electrophoresis, blocked with 20% skim milk and incubated with primary antibody overnight at 4 °C. Western blotting was performed using an enhanced chemiluminescence detection reagent (Beyotime Biotechnology Inc., Shanghai, China.) and according to the manufacturer’s protocol. *P* < 0.05 was considered statistically significant.

### Statistical analysis

All statistical analyses were performed using the SPSS version 21.0 software package (SPSS, Chicago, Il, USA). The data are expressed as mean ± standard deviation. Categorical variables were analyzed using the χ2 or Fisher’s exact test. For paired samples, continuous variables were analyzed using the student’s t-test, and differences between groups were analyzed using an analysis of variance calculation. When the basic assumptions of the student’s t-test were not satisfied, the Wilcoxon–Mann–Whitney test was used. *P*-value < 0.05 was considered to indicate a statistically significant difference.

## Results

### Identification of DEGs

**Figure 1:**
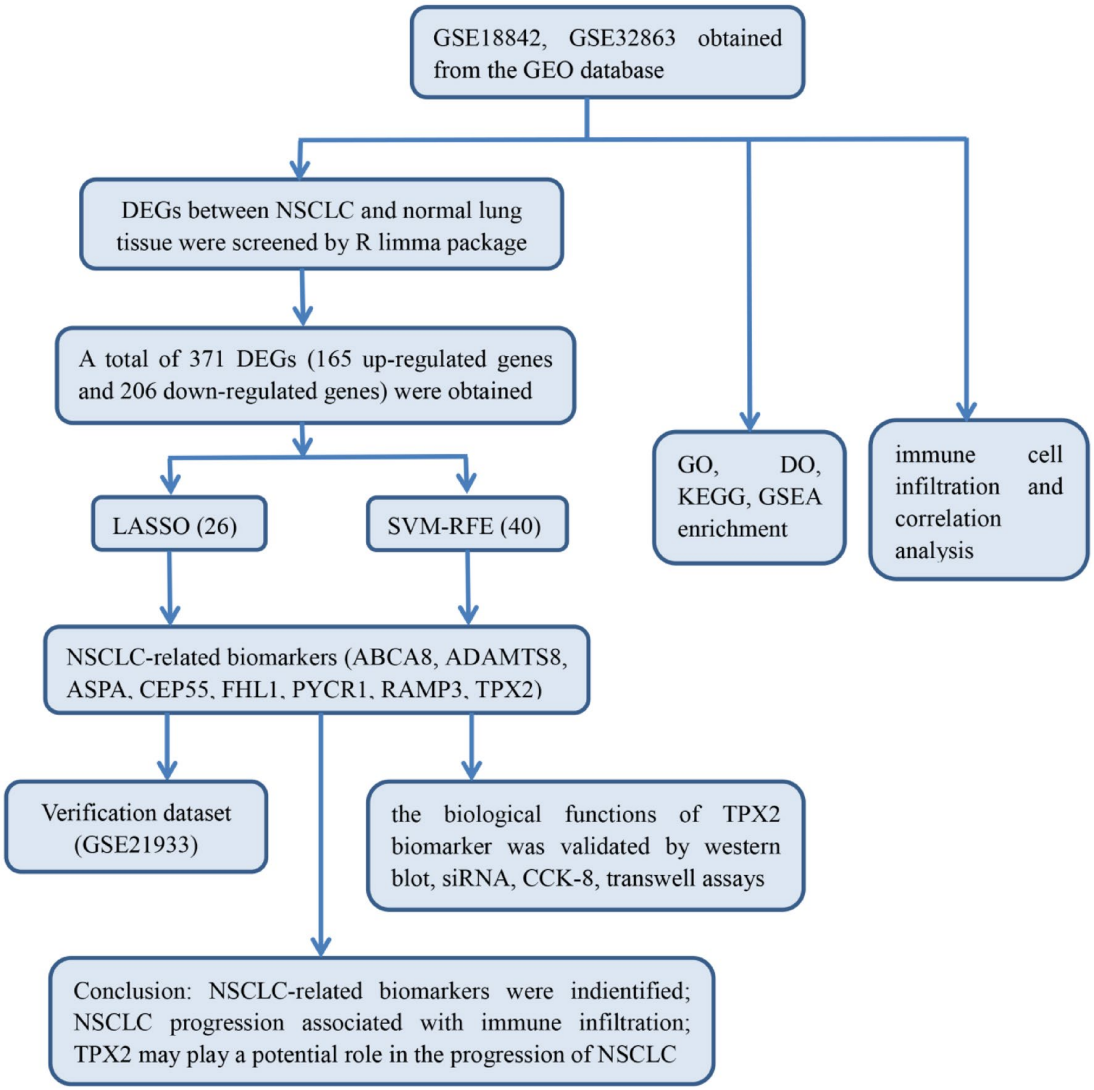
a flowchart of the entire analysis process to the manuscript.

**Figure 2:**
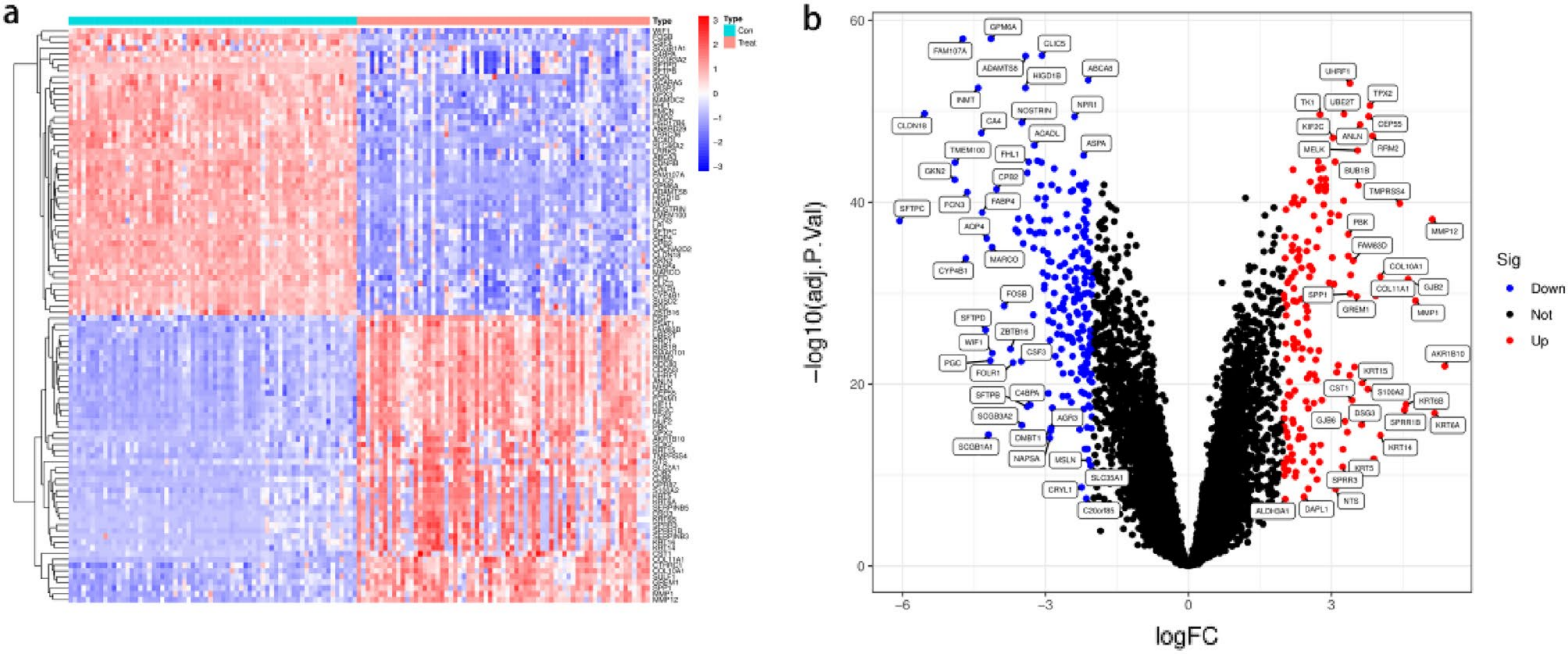
Identification of DEGs. (**a**) Heatmap of DEGs. (**b**) Volcano diagram of DEGs, red indicates up-regulated, blue indicates down-regulated.

To analyze the diagnostic genes of NSCLC, we designed a flowchart (Fig. 1). Using the ‘Limma’ package in the R language, 371 DEGs, including 165 up-regulated genes and 206 down-regulated genes, were screened in NSCLC and normal lung tissue samples of GSE18842, GSE32864, and GSE21933 according to the criteria (adjusted *P*-value < 0.05 and |logFC|≥ 1). The results were expressed in the heatmap (Fig. 2a) and a volcano plot (Fig. 2b).

### Functional enrichment analyses of the DEGs

**Figure 3:**
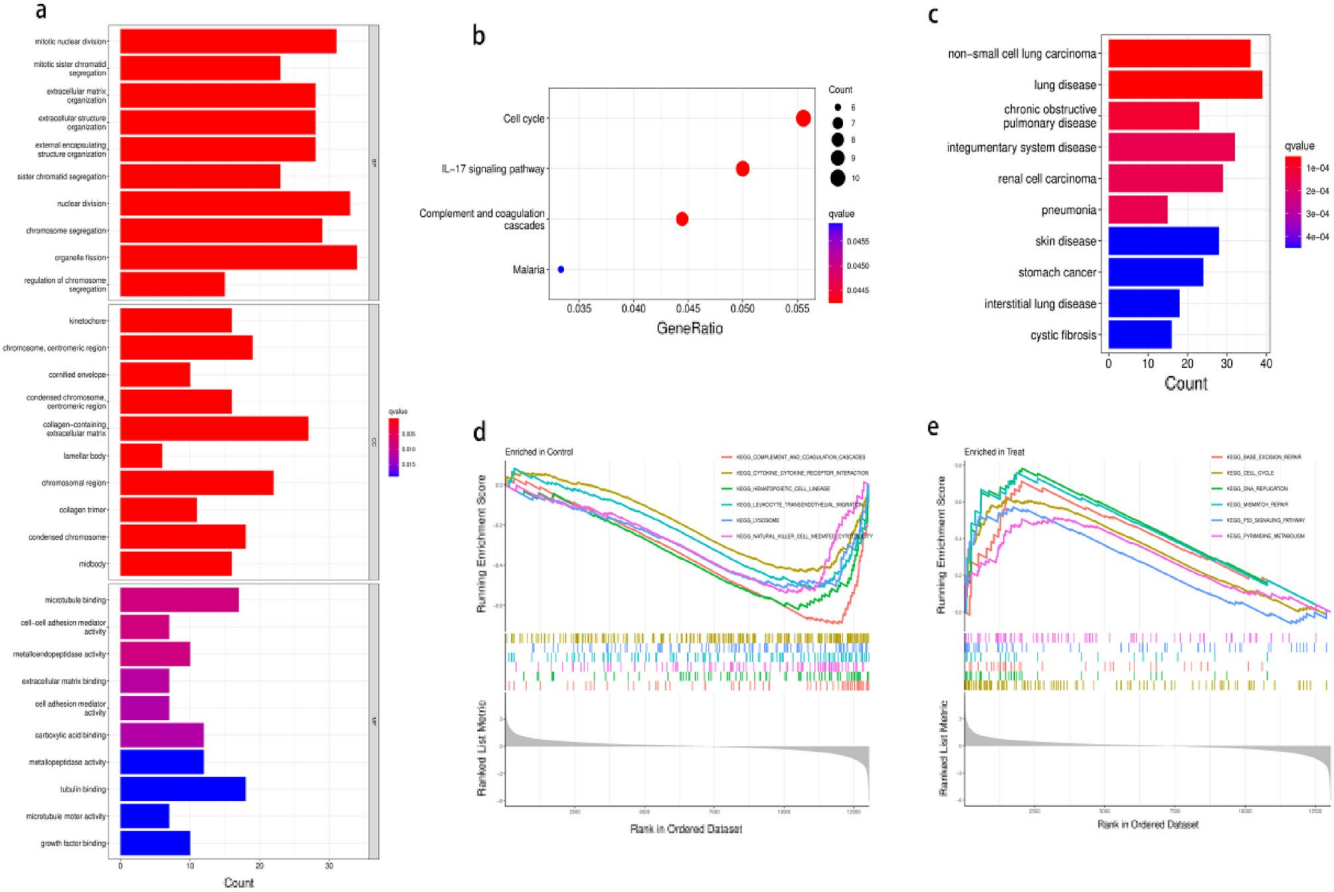
Functional enrichment analysis of DEGs. The *P* value represents the colour depth of the node. The size of the node implies the number of DEGs. (**a**) GO functional enrichment analysis results for DEGs, including Biological process(BP), molecular function(MF) and cellular component(CC). (**b**) KEEG enrichment analysis reveals signalling pathways highly relevant to NSCLC. (**c**) DO functional enrichment analysis results of DEGs. (**d**) GSEA shows the top six signalling pathways most associated with normal lung tissue. (**e**) GSEA shows the top six signalling pathways most associated with NSCLC.

We investigated the possible biological functions of these 371 DEGs using the GO, KEGG, DO, and GSEA functional enrichment analyses. The GO analysis revealed that DEGs were primarily enriched in nuclear division and extracellular matrix organization, implying a link between tumor cell division and distant tumor metastasis (Fig. 3a). According to KEGG pathway analysis, the IL-17 signaling pathway, cell cycle, complement and coagulation cascades, and malaria were four significantly enriched pathways (Fig. 3b). The DO analysis revealed that these DEGs were remarkably enriched in lung disease, NSCLC, and integumentary system disease (Fig. 3c). Finally, the GSEA analysis revealed that in lung cancer tissue, complement and coagulation cascades, cytokine-cytokine receptor interactions, hematopoietic cell lineage, leukocyte transendothelial migration, lysosome, and natural killer cell-mediated cytotoxicity were highly active (Fig. 3d), whereas base excision repair, cell cycle, DNA replication, mismatch repair, p53 signaling pathway, and pyrimidine metabolism were highly active in normal nasopharyngeal tissue (Fig. 3e). All these findings indicate that these DEGs may be critical in NSCLC.

### Screening for NSCLC biomarkers and validation

**Figure 4:**
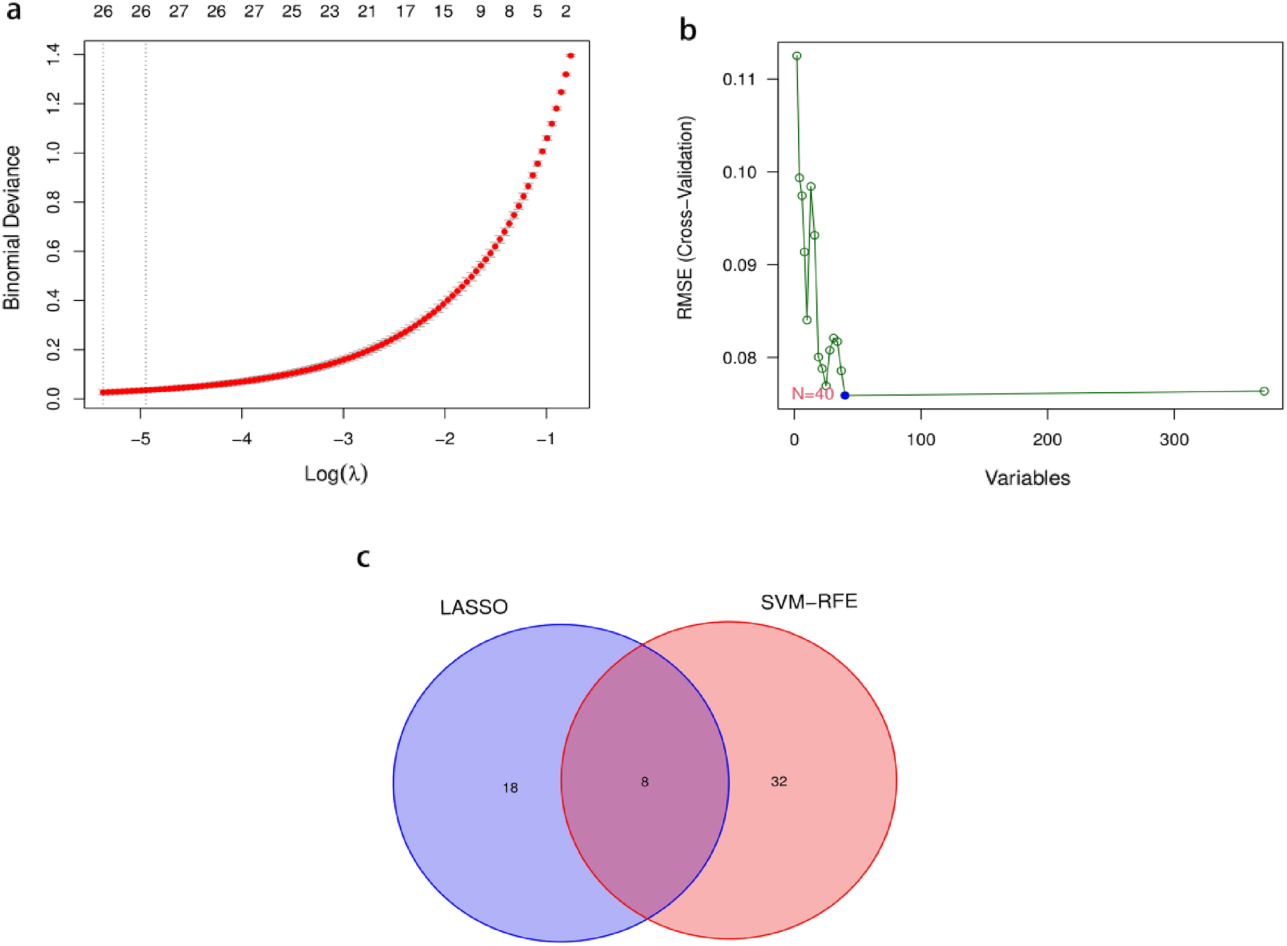
Machine learning approach to screen for NSCLC-related biomarkers. The point corresponding to the smallest vertical coordinate is the characteristic genes. (**a**) Results of screening biomarkers based on LASSO algorithm. (**b**) Screening results for biomarkers based on the SVM-RFE algorithm. (**c**) The Venn diagram shows the results of the intersection of the LASSO algorithm and the SVM-RFE algorithm, with the intersection resulting in eight biomarkers.

**Figure 5:**
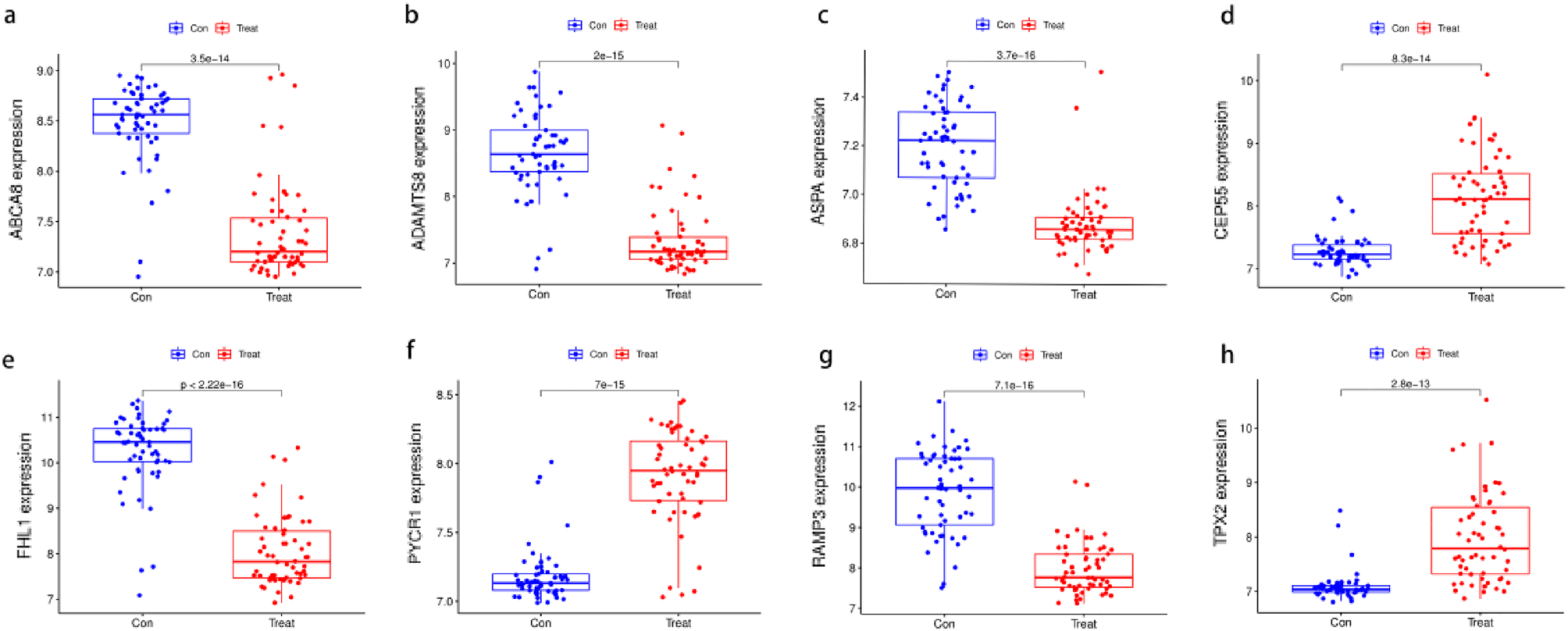
Expression of eight biomarkers in the validation group (GSE32863). (**a**) ABCA8. (**b**) ADAMTS8. (**c**) ASPA. (**d**) CEP55. (**e**) FHL1. (**f**) PYCR1. (**g**) RAMP3. (**h**) TPX2. *P* < 0.05 means difference is statistically significant.

**Figure 6:**
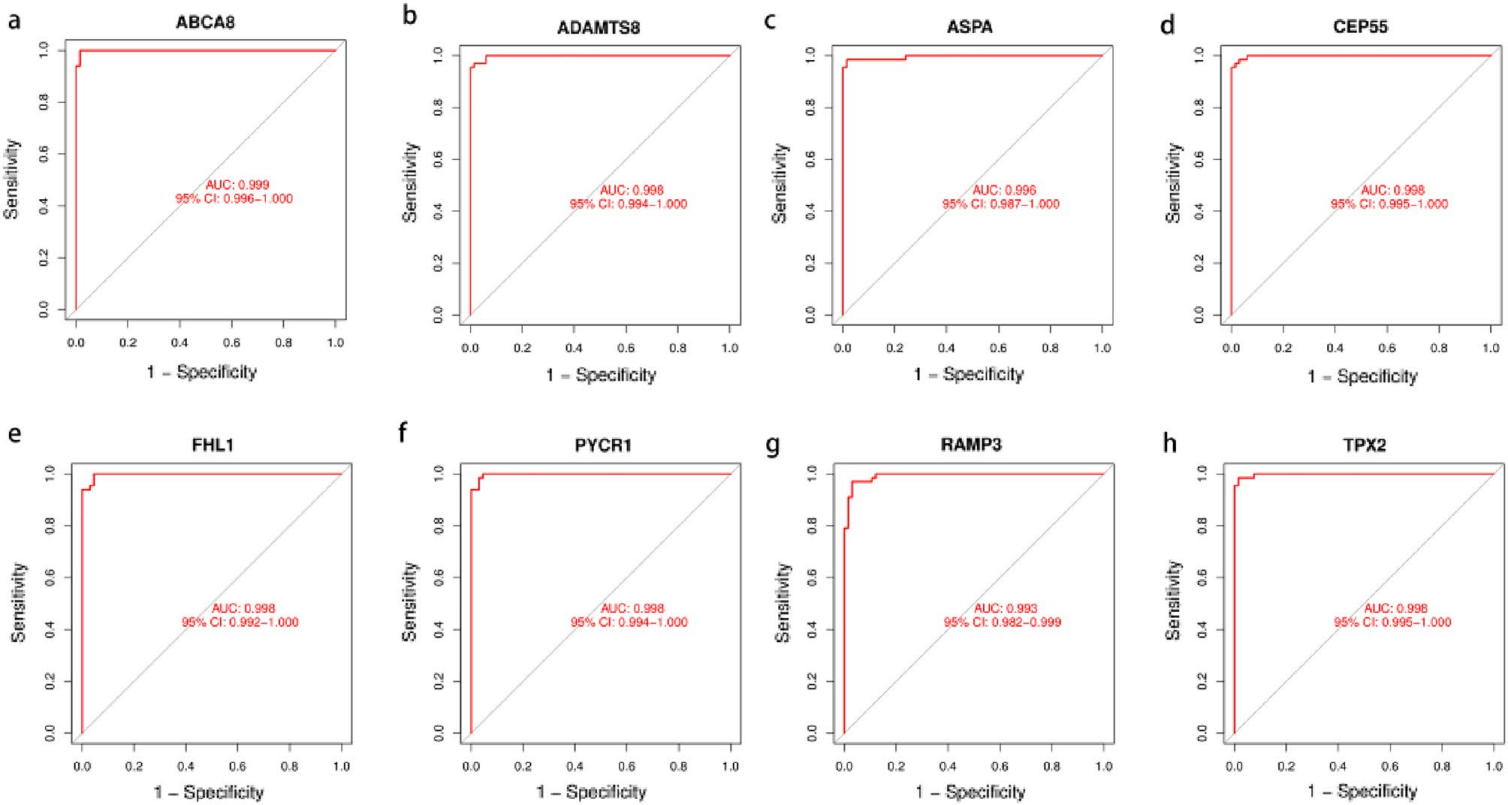
ROC curves for eight biomarkers in the training dataset(GSE18842 and GSE21933). (**a**) ABCA8. (**b**) ADAMTS8. (**c**) ASPA. (**d**) CEP55. (**e**) FHL1. (**f**) PYCR1. (**g**) RAMP3. (**h**) TPX2.

**Figure 7:**
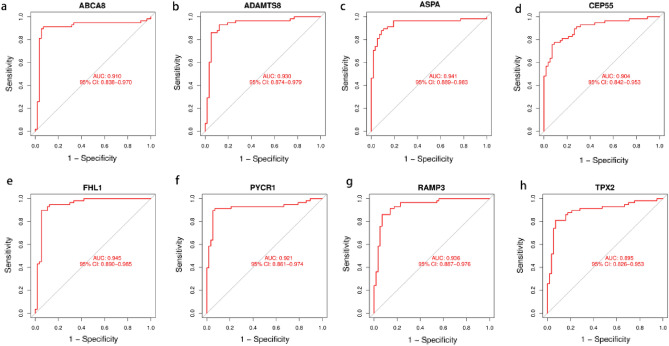
ROC curves for eight biomarkers in the validation dataset (GSE32863). (**a**) ABCA8. (**b**) ADAMTS8. (**c**) ASPA. (**d**) CEP55. (**e**) FHL1. (**f**) PYCR1. (**g**) RAMP3. (**h**) TPX2.

The LASSO logistic algorithm was used in this study to identify, 26 characteristic genes (Fig. 4a), while the SVM-RFE method was used to identify 40 characteristic genes (Fig. 4b). Eight biomarker genes were obtained as a result of the intersection, *ABCA8*, *ADAMTS8*, *ASPA*, *CEP55*, *FHL1*, *PYCR1*, *RAMP3*, and *TPX2* genes (Fig. 4c). To further validate their potential as diagnostic biomarkers for NSCLC, we examined their expression in the GSE32863 dataset (Fig. 5), which revealed that *ABCA8*, *ADAMTS8*, *ASPA*, *FHL1*, and *RAMP3* genes were down-regulated in NSCLC while *CEP55*, *PYCR1*, and *TPX2* genes were up-regulated. The accuracy of these eight biomarkers in distinguishing NSCLC from normal individuals was evaluated using a receiver operating characteristic (ROC) analysis, and all eight biomarkers demonstrated high sensitivity and specificity. (The areas under the ROC curves (AUCs) = 0.999 in GSE18842 and GSE 21993 and 0.910 in GSE32863 for the *ABCA8* gene; AUCs = 0.998 in GSE18842 and GSE21993 and 0.930 in GSE32863 for the *ADAMTS8* gene, AUCs = 0.996 in GSE18842 and GSE 21993 and 0.941 in GSE32863 for the *ASPA* gene, AUCs = 0.998 in GSE18842 and GSE21993 and 0.904 in GSE32863 for the *CEP55* gene, AUCs = 0.998 in GSE18842 and GSE21993 and 0.945 in GSE32863 for the *FHL1* gene, AUCs = 0.998 in GSE18842 and GSE21993 and 0.921 in GSE32863 for the *PYCR1* gene, AUCs = 0.993 in GSE18842 and GSE21993 and 0.936 in GSE32863 for the *RAMP3* gene, AUCs = 0.998 in GSE18842 and GSE21993 and 0.895 in GSE32863 for the *TPX2* gene. All *P* < 0.05) (Figs. 6 and 7).

### Assessment of immune cell infiltration

**Figure 8:**
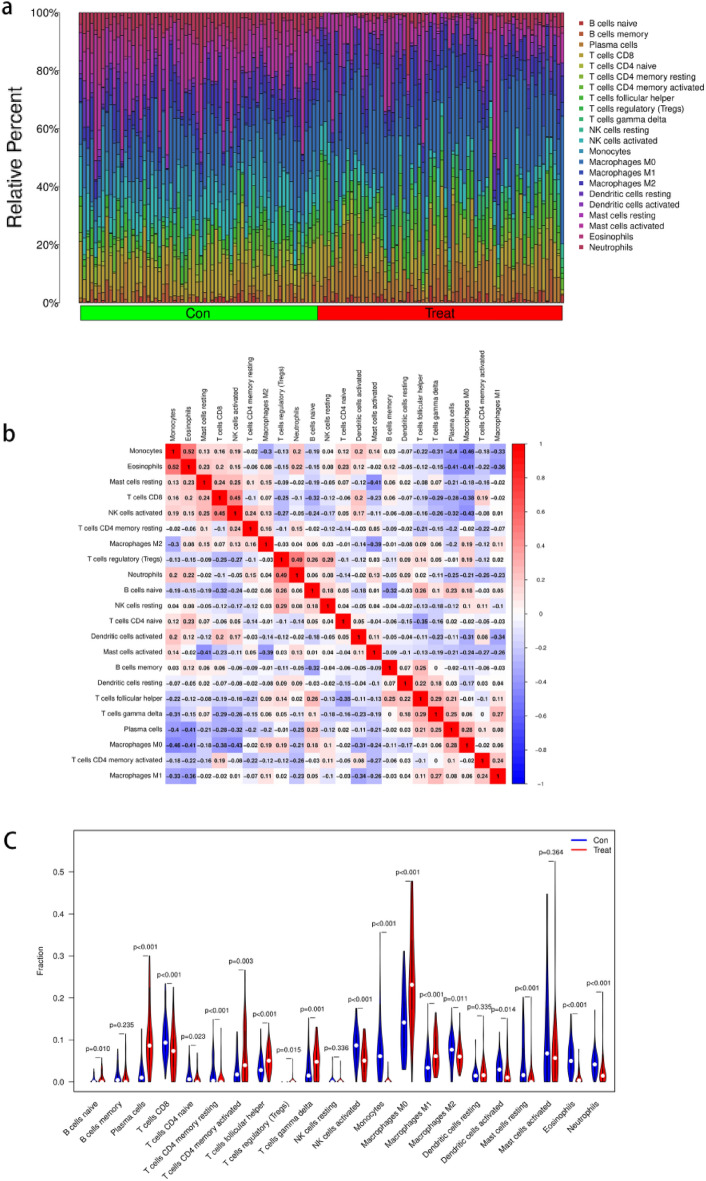
Correlation analysis among immune cells and Differential analysis of immune cells. (**a**) The 22 immune cell populations in normal lung tissue and NSCLC. ‘con’ represents normal lung tissue, ‘treat’ represents NSCLC. The vertical coordinate represents the amount of immune cells. (**b**) Correlation heatmap Indicates the correlation analysis of immune cells. The redder of the point, the stronger the positive correlation, the reddest point represents the two immune cells with the most significant positive correlation. The bluer the colour of the point, the stronger the negative correlation, the two immune cells with the most significant negative correlation correspond to the bluest points. (**c**) The vioplot showed the difference in 22 immune cells between normal lung tissue and NSCLC. ‘con’ represents normal lung tissue, ‘treat’ represents NSCLC. *P* < 0.05 means difference is statistically significant.

**Figure 9:**
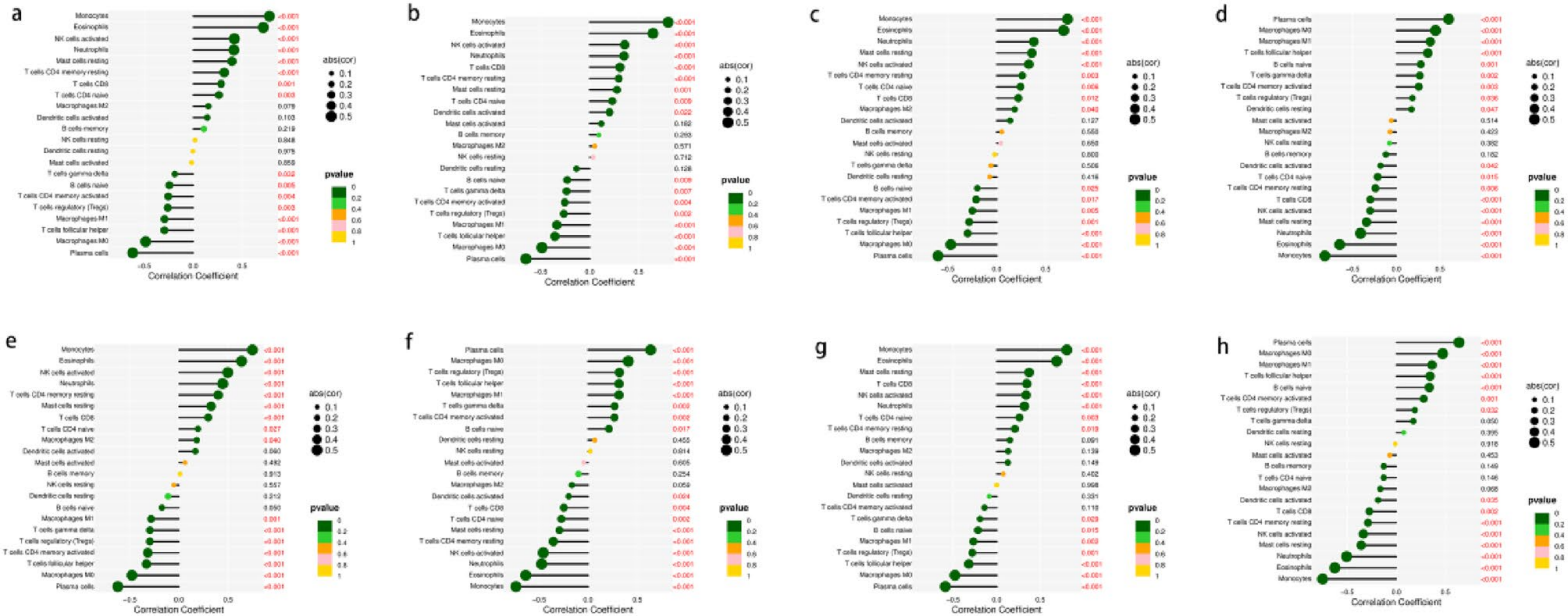
Correlation analysis between eight biomarkers and immune cells. Colours represent *P*-values, *P* < 0.05 means significant correlation and is indicated in red, the size of the circle represents the absolute value of the correlation coefficient. (**a**) ABCA8. (**b**) ADAMTS8. (**c**) ASPA. (**d**) CEP55. (**e**) FHL1. (**f**) PYCR1. (**g**) RAMP3. (**h**) TPX2.

A comprehensive and dynamic understanding of the immune microenvironment is essential to develop effective therapeutic strategies. Therefore, in this study, we investigated immune cell infiltration in NSCLC and the relationship between biomarkers and infiltrating immune cells. First, we found significant differences in the composition of the 22 infiltrating immune cell types in each tissue sample (Fig. 8a). The correlation matrix showed the strongest positive correlation between eosinophils and monocytes and the strongest negative correlation between macrophage M0 cells and monocytes (Fig. 8b). Monocytes and eosinophils were the most down-regulated cells in NSCLC, while plasma cells and macrophage M0 were the most up-regulated, and activation of neutrophils, macrophage M1, and NK cells was low (Fig. 8c). Figure 8 depicts the relationship between the expression of eight biomarkers and the infiltration of immune cells. Monocytes, eosinophils, NK cells activated, neutrophils, mast cells resting, and T cells CD4 memory resting were positively correlated with with the ABCA8 gene expression, whereas plasma cells, macrophages M0, Follicular helper T (Tfh) cells, and macrophages M1 were negatively correlated (Fig. 9). The ADAMTS8 expression levels were negatively correlated with macrophages M1 T cells follicular helper, macrophages M0, and plasma cells, and positively correlated with monocytes, eosinophils, NK cells activated, neutrophils, T cells CD8, and T cells CD4 memory resting. The with the APSA gene expression levels expression levels were negatively correlated with T cells follicular helper, macrophages M0, and plasma cells and positively correlated with monocytes, eosinophils, neutrophils, mast cells resting, and NK cells activated. With The CEP55 gene expression levels expression levels were positively correlated with plasma cells, macrophages M0, macrophages M1, and T cells follicular helper and negatively correlated with T cells CD8, NK cells activated, mast cells resting, neutrophils, eosinophils, and monocytes. With The FHL1 gene expression levels expression levels were positively correlated with monocytes, eosinophils, NK cells activated, neutrophils, T cells CD4 memory resting, mast cells resting, and T cells CD8 and negatively correlated with T cells gamma delta, T cells regulatory, T cells CD4 memory activated, T cells follicular helper, macrophages M0, and plasma cells. With the PYCR1 gene expression levels were positively correlated with plasma cells, macrophages M0, T cells regulatory, T cells follicular helper, and macrophages M1 and negatively correlated with mast cells resting, T cells CD4 memory resting, NK cells activated, neutrophils, eosinophils, and monocytes. With The RAMP3 gene expression levels were positively correlated with monocytes, eosinophils, mast cells resting, T cells CD8, NK cells activated, and neutrophils, and negatively correlated with T cells follicular helper, macrophages M0, and plasma cells. The TPX2 expression levels were positively correlated with plasma cells, macrophages M0, macrophages M1, T cells follicular helper, and B cells naive and negatively correlated with T cells CD4 memory resting, NK cells activated, mast cells resting, neutrophils, eosinophils, and monocytes (*P* < 0.01).

### Survival analysis

**Figure 10:**
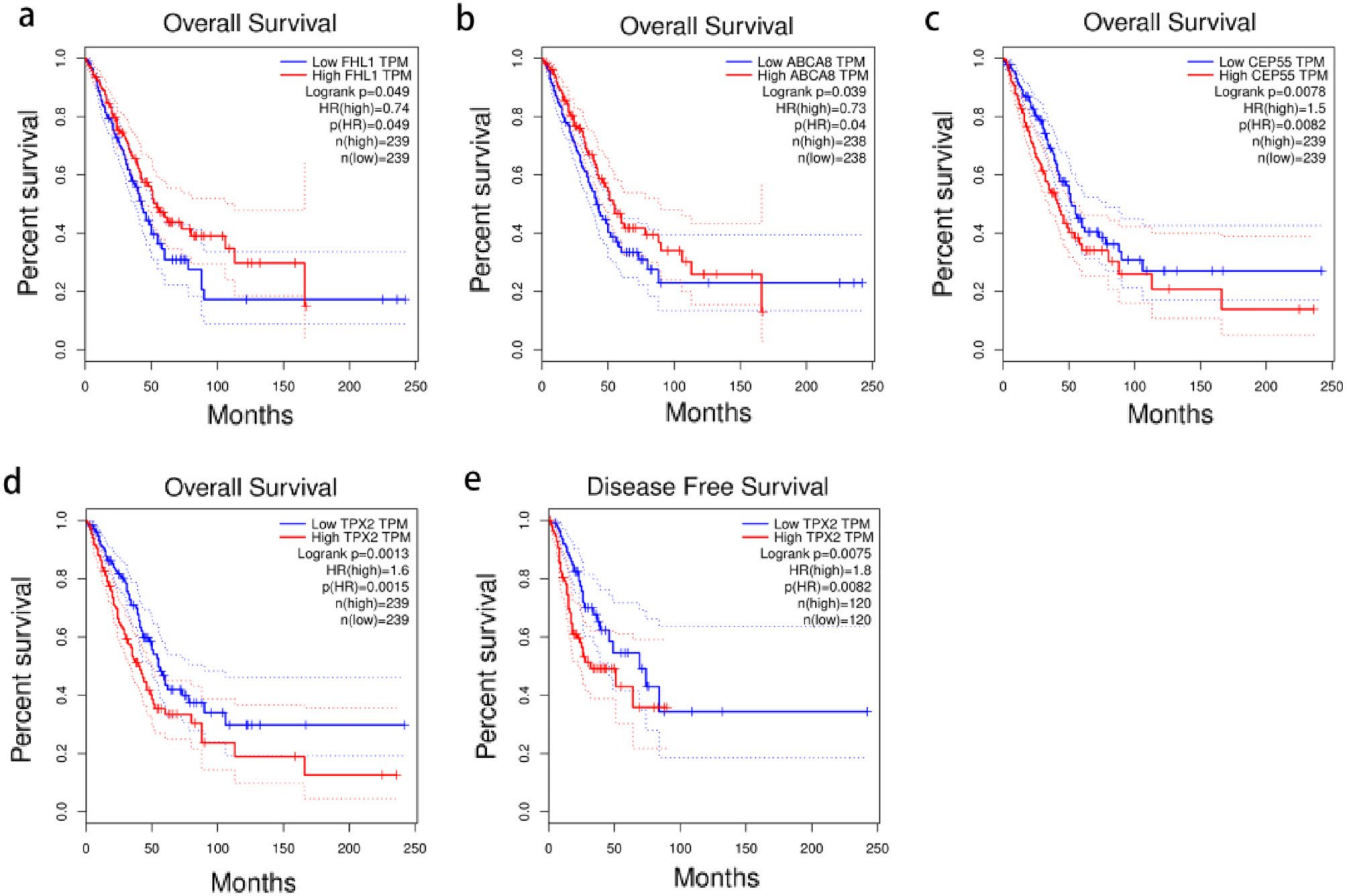
Survival analysis of eight biomarkers in GEPIA website. (**a–d**) Overall survival analysis of FLHI, ABCA8, CEP55, TPX2. (**e**) Disease Free survival analysis of TPX2. *P* < 0.05 means difference is statistically significant.

To determine the potential prognostic value of these eight biomarkers, we investigated the relationship between each biomarker’s expression and OS and DFS in NSCLC. The findings revealed that *ABCA8* and *FHL1* genes were associated with longer OS in lung cancer patients (*P* < 0.05), whereas *TPX2* and *CEP55* genes were associated with shorter OS (*P* < 0.05) (Figs. 10a–d), and other biomarkers were not significantly associated with OS (*P* > 0.05). Notably, only the *TPX2* gene was associated with poor DFS (Fig. 10e), suggesting that the *TPX2* gene may have a potential prognostic value in NSCLC, which we investigated further.

### Knockdown of the TPX2 gene inhibited the proliferation and migration of A549 cells

**Figure 11:**
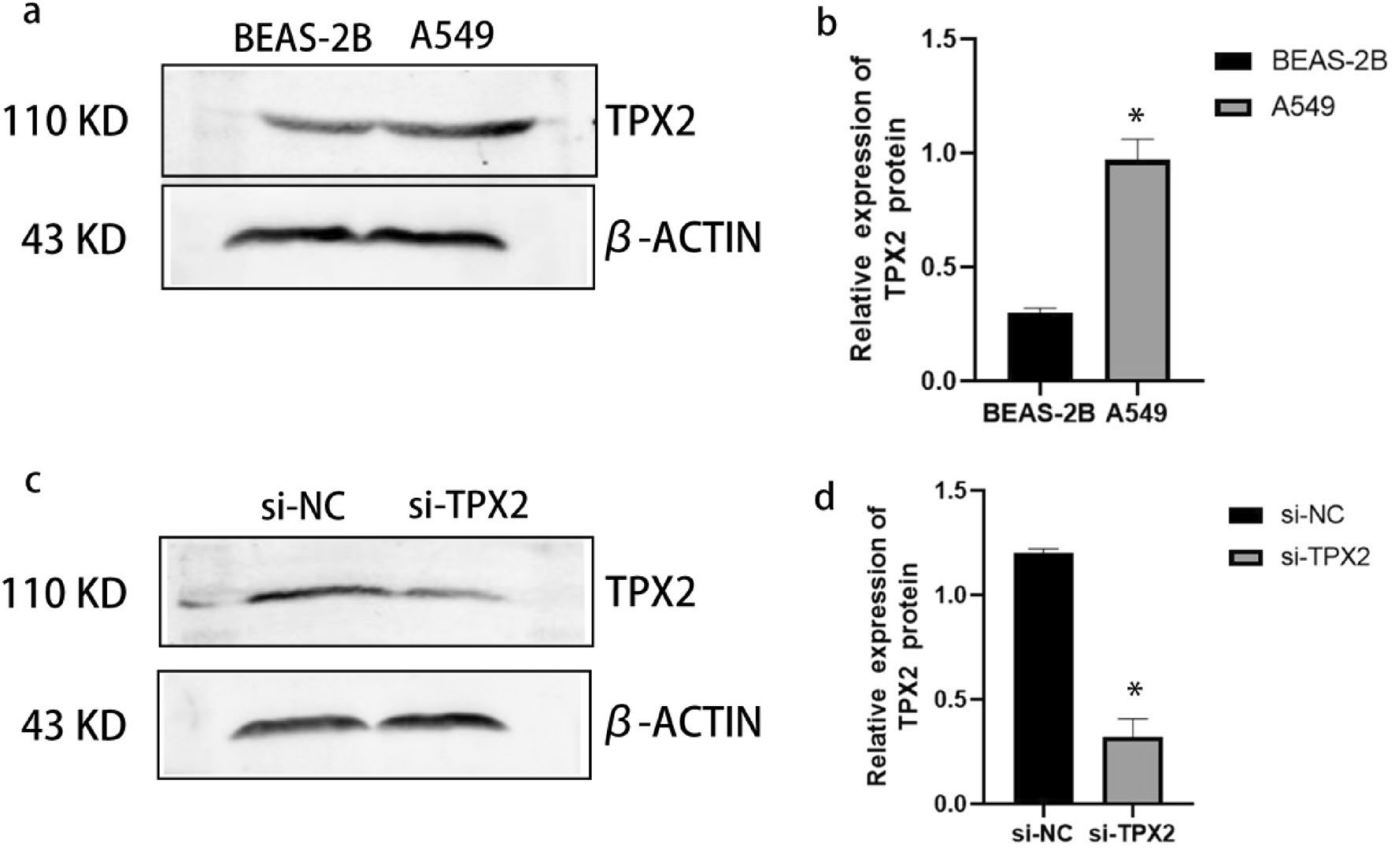
(**a, b**) Western blot method to detect TPX2 protein expression in normal alveolar epithelial cells BEAS-2B and non-small cell lung cancer cells A549. (**c, d**) Western blot method to detect si-TPX2 transfection efficiency. ‘*’represents *P* < 0.05.

**Figure 12:**
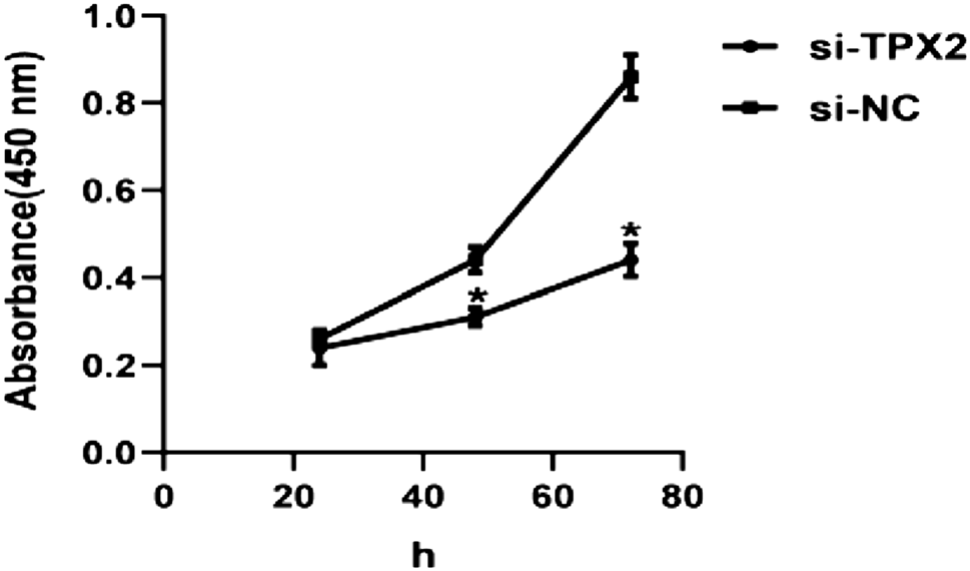
CCK8 method to detect the proliferative ability of the si-NC and si-TPX2 groups. Silencing of TPX2 inhibited the proliferative capacity of A549 cells. ‘*’ represents *P* < 0.05.

**Figure 13:**
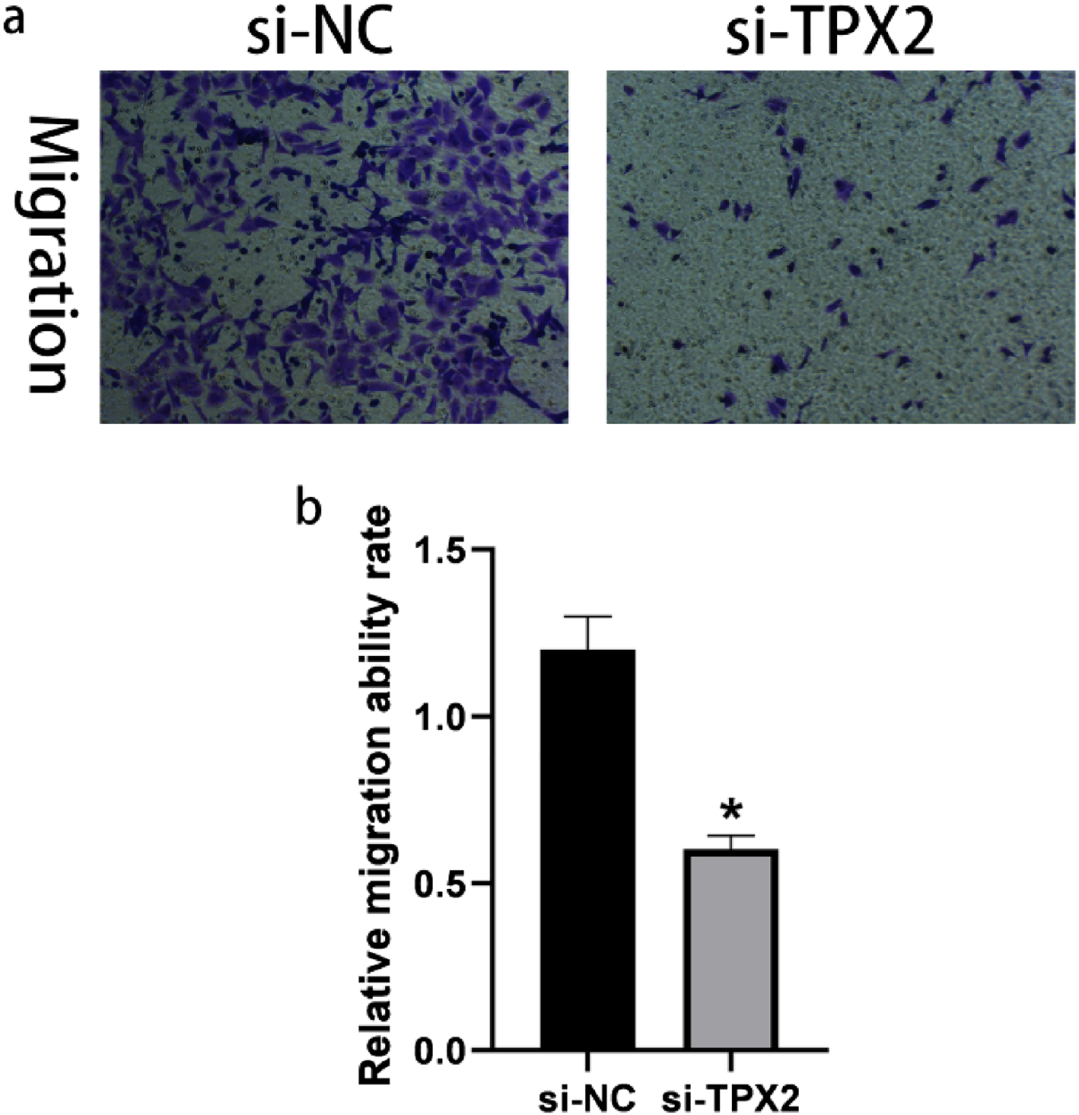
Transwell method to detect migration ability of si-NC group and si-TPX2 group. Silencing of TPX2 suppressed migration ability in A549 cells. ‘*’ represents *P* < 0.05.

To investigate the potential role of the *TPX2* gene in NSCLC, we used a western blot to compare the expression of TPX2 in A549 cells and BEAS-2B cells. We found that A549 cells had higher levels of TPX2 protein expression than BEAS-2B cells (*P* < 0.05) (Fig. 11a, b). Furthermore, we inhibited TPX2 expression by transfecting si-RNA-targeted TPX2 into A549 cells. The results of western blot analysis revealed that the levels of TPX2 proteins were significantly lower in A549 cells after transfection with si-TPX2 compared to si-control (*P* < 0.05) (Fig. 11c, d), indicating that the transfection was complete and ready for the next step of the experiment. The CCK8 proliferation assays confirmed that the *TPX2* gene knockdown significantly inhibited A549 cell proliferation (*P* < 0.05) (Fig. 12). Moreover, the results of the transwell migration assay revealed that the relative number ratios of migrating cells were significantly lower in the *TPX2* gene knockdown cells compared to si-control cells (*P* < 0.05) (Fig. 13), indicating that the *TPX2* gene knockdown resulted in A549 cell migration ability. Therefore, we speculated that the *TPX2* gene might act as an oncogene, promoting NSCLC progression. However, this conclusion needs to be verified via in vivo experiments.

## Discussion

NSCLC is well known for being asymptomatic and can only be detected at an early stage through physical examination^1^. As the tumor grows, develops, and spreads, serious symptoms emerge, including chest pain, breathing difficulties, liver metastases, and a slew of seriously life-threatening symptoms. Given the lack of obvious symptoms in the early stages of NSCLC, which makes diagnosis difficult, “early diagnosis and early treatment” has become the treatment consensus for NSCLC^31^. With the advancement of bioinformatics, effective analysis and exploration of cancer genes to find tumor biomarkers have become a hot topic for early cancer diagnosis expression profiles and treatment, For example, chen et al. have used new computational models in the field of miRNA to make great contributions to the pathogenesis of diseases and new drug development. For example, through the construction of Neighborhood Constraint Matrix Completion for MiRNA-Disease Association prediction (NCMCMDA), deep-belief network for miRNA-disease association prediction (DBNMDA), Ensemble of Decision Tree based MiRNA-Disease Association prediction (EDTMDA) models to accurately predict the potential relationship between miRNA-disease and in breast neoplasms, lung neoplasms, esophageal neoplasms have been validated^32–34^. The study of chen et al. has greatly improved the experimental efficiency and provided a new theoretical basis for the prevention, diagnosis and treatment of complex human diseases by screening disease-associated miRNAs through computational models, but such methods have not been adequately studied and described for biomarkers of NSCLC progression. Therefore, it is critical to identify a sensitive, safe, and feasible NSCLC biomarker for diagnostic and therapeutic purposes and to improve patient survival^35^.

The bioinformatics analysis of the microarray dataset from the GEO database identified 165 up-regulated and 206 down-regulated genes between NSCLC and normal lung tissue samples. Moreover, functional analyses revealed that these DEGs were linked to lung cancer tumorigenesis and metastasis. The most significantly enriched pathway, the cell cycle signaling pathway, has been shown to have mutations that can affect the genomic and microenvironmental characteristics of LUAD patients^36^ and can also be used as an assessment criterion for post-operative adjunctive therapy in LUAD patients^37^. Furthermore, GSEA analysis revealed that these DEGs might affect base excision repair, the cell cycle, DNA replication, mismatch repair, and the p53 signaling pathway. The most activated p53 signaling pathway, which has been linked to oncogenic effects^38^, promotes tumor growth in NSCLC and pancreatic ductal adenocarcinoma (PDAC)^39,40^. However, these DEGs’ specific functions and molecular mechanisms need to be investigated further.

Because of its flexibility and power, ML is increasingly being used to screen novel biomarkers. We used two recently popular ML approaches, LASSO logistic regressions, and SVM-RFM, to identify the best diagnostic biomarkers for NSCLC. After combining the two methods and ROC analysis, eight NSCLC-related biomarkers with accurate predictive properties were identified (the AUCs of all these eight genes were greater than 0.89), including *ADAMTS8*, *ABCA8*, *TPX2*, *CEP55*, *ASPA*, *FHL1*, *RAMP3*, and *PYCR1* genes. A disintegrin and metallopeptidase with thrombospondin motif type 8 (ADAMTS8) is a member of the zinc metalloproteinase family and is considered a tumor suppressor^41^. Wu et al. found that ADAMTS8 has been associated with clinical staging and lymph node metastasis in esophageal cancer patients^42^. ADAMTS8 can also inhibit lung cancer by targeting Vascular endothelial growth factor (VEGFA)^43^. ATP-binding cassette subfamily A 8 (ABCA8) has been linked to tumors. Studies have shown that ABCA8 can inhibit the proliferation of breast cancer cells by regulating the AMPK/mTOR signaling pathway^44^, and ABCA8 can also be used as a prognostic marker for hepatocellular carcinoma^45^ and gastric adenocarcinoma^46^. Centrosomal protein, 55 kD (CEP55) has been reported to be a potential biomarker and therapeutic target for PDAC and lung cancer^47^. Several studies have shown that CEP55 is carcinogenic in the colon and esophageal cancers^48,49^. Targeting protein for xenopus kinesin-like protein 2 (TPX2) is a cell cycle-associated gene that plays a pro-oncogenic role in hepatocellular carcinoma cells and acts synergistically with anti-cancer drugs^50^. TPX2 can also be a therapeutic target for breast cancer and is associated with patient prognosis^51^. Current studies have shown that recombinant *Helicobacter Pylori* aspartate ammonia-lyase (ASPA) is an effective predictor of prognosis in colorectal cancer patients^52^. Four-and-a-half LIM domains protein (FHL1) has a diagnostic value in microscopic papillary thyroid carcinoma^53^, and it has been reported that FHL1 acts as an inhibitor in colorectal cancer cells^54–56^. Receptor activity-modifying protein 3 (RAMP3) is an accessory molecule that forms complexes with and regulates the function of specific G protein-coupled receptors (GPCRs). To date, studies have shown that RAMP3 is overexpressed in hepatocellular carcinoma patients and that RAMP3 is an independent prognostic factor for overall survival and RFS^57^. Pyrroline-5-Carboxylate Reductase 1 (PYCR1) is a mitochondrial enzyme that is the final step in the proline biosynthetic pathway. Currently, PYCR1 has been reported to regulate tumour cell proliferation in hepatocellular carcinoma and cloud be an effective therapeutic target for multiple myeloma^58,59^, as well as inhibit the development of clear cell renal cell carcinoma by causing mitochondrial dysfunction and interfering with oxidative stress pathways^60^. Thus, according to the available studies, the eight biomarkers mentioned above play an important role in tumorigenesis and progression, but their exact mechanisms in NSCLC remain unknown.

Given the importance of the immune microenvironment in the development of lung cancer, we also performed immunological analyses. We found that monocytes differed most between normal and NSCLC tissue samples and correlated closely with all eight previously obtained biomarkers, implying that monocytes may be the most active immune cells in NSCLC. Monocytes have been shown to play a role in tumor progression^61^. For example, up-regulation of monocytes promotes CT26 tumor progression^62,63^. It has also been linked to the immune checkpoint matrix metalloproteinases in hepatocellular carcinoma^64^. The findings of this study suggest that monocytes may contribute to clinical immunotherapy and warrant further investigation.

Another significant finding in our study was that TPX2 expression was linked to poor prognosis, and TPX2 was up-regulated in both bioinformatics and western blot validation. We also found that TPX2 promoted the proliferation and migration of lung cancer A549 cells in vitro, suggesting that TPX2 was involved in developing NSCLC and could be a therapeutic target for NSCLC. However, the exact mechanism remains unknown, and we will investigate the role of TPX2 in vivo experiments.

ML methods have been successfully applied in cancer research in recent years, for example, for cancer classification^65^, for analyzing gene chip data to screen for cancer-related biomarkers to assist doctors in making better diagnoses and decisions^66^, and for cancer prediction to significantly improve prediction accuracy^65^. Notably, the ML method has been demonstrated to be effective in determining a non-invasive, accurate, and reliable diagnosis of NSCLC. Li et al. used specific carbonyl volatile organic compounds in exhaled breath as a biomarker for detecting lung cancer to distinguish lung cancer patients from healthy controls and patients with benign lung nodules^66^. Zhang et al. suggest that 5-hydroxymethylcytosine in circulating cell-free DNA can be used to diagnose and treat NSCLC^67^. Zhang et al. concluded that five circulating micro RNAs have important prognostic capabilities in lung cancer^68^. Wang et al. identified eight differentially expressed long non-coding RNAs in LUAD that could be used as potential diagnostic biomarkers^69^.

This study combines machine learning methods (LASSO algorithm and SVM-RFE algorithm) with medical experiments to identify non-small cell lung cancer (NSCLC)-related biomarkers through the GEO public database, and finds that the progression of NSCLC is associated with immune cell infiltration, which greatly improves the efficiency of basic experiments for clinicians, and the screened NSCLC-related biomarkers is important for the prevention, diagnosis and treatment of NSCLC. Subsequently, we used in vitro functional assays to silence the prognostic TPX2 biomarker gene in NSCLC A549 cells and found that the proliferation and migration ability of A549 cells were significantly reduced, which further confirmed that the NSCLC-related biomarkers screened by the machine learning approach are novel and reliable. Our study provides a new idea for the pathogenesis and targeted therapy of NSCLC.

## Conclusion

In conclusion, we used machine learning methods to identify eight diagnostic biomarkers for NSCLC, including *ADAMTS8*, *ABCA8*, *TPX2*, *CEP55*, *ASPA*, *FHL1*, *RAMP3*, and *PYCR1* genes, followed by functional enrichment analysis and immune correlation analysis, and we validated the potential role of the *TPX2* gene in vitro. Our findings identify new potential biomarkers for diagnosing and treating NSCLC and reveal new approaches that may have therapeutic potential for NSCLC.

## Supplementary Information

Supplementary Information.

## Data Availability

The datasets generated during the current study are available in the Gene Expression Omnibus (GEO) repository, (https://www.ncbi.nlm.nih.gov/geo/), (https://www.ncbi.nlm.nih.gov/geo/query/acc.cgi?acc=GSE18842), (https://www.ncbi.nlm.nih.gov/geo/query/acc.cgi?acc=GSE21933), (https://www.ncbi.nlm.nih.gov/geo/query/acc.cgi?acc=GSE32863).
