# Supplementary material for: Identidication of novel biomarkers in non-small cell lung cancer using machine learning
Source: Sci Rep. 2022 Oct 6;12:16693. doi: 10.1038/s41598-022-21050-5 (PMC9537298; doi:10.1038/s41598-022-21050-5)
Supplement: Supplementary file 1 — Supplementary Information. [file 41598_2022_21050_MOESM1_ESM.pdf]

1.

BEAS-2B A549

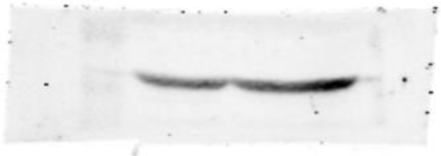

TPX2(110bp)

BEAS-2B A549

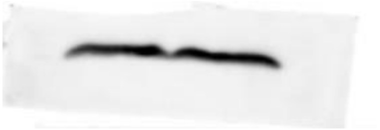

actin(43bp)

2.

BEAS-2B A549

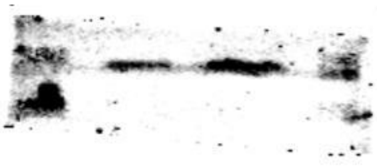

TPX2(110bp)

BEAS-2B A549

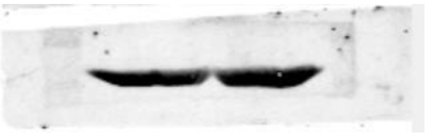

β-actin(43bp)

3.

BEAS-2B A549

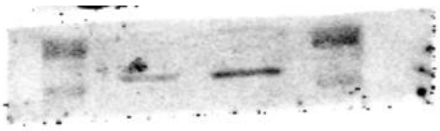

TPX2(110bp)

BEAS-2B A549

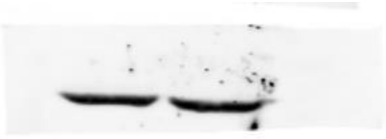

actin(43bp)

Fig. S1. Repeat image of TPX2 in wb three times .(Corresponding to Fig 10a)

1.

si-NC si-TPX2

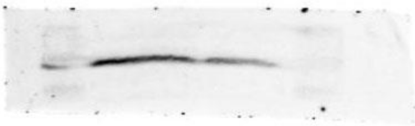

TPX2(110bp)

si-NC si-TPX2

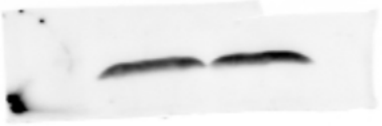

β-actin(43bp)

2.

si-NC si-TPX2

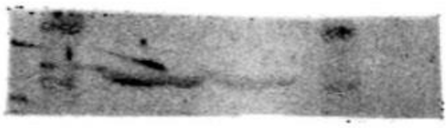

TPX2(110bp)

si-NC si-TPX2

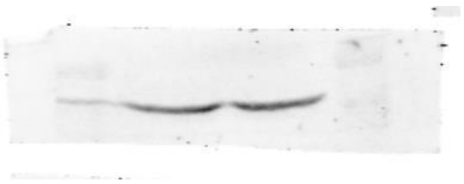

β-actin(43bp)

3.

si-NC si-TPX2

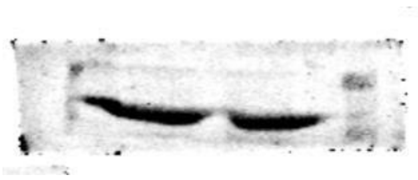

TPX2(110bp)

si-NC si-TPX2

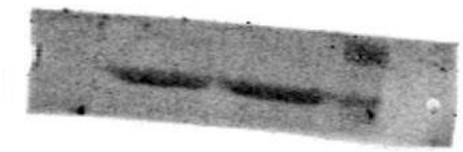

β-actin(43bp)

Fig. S2. Repeat image of TPX2 in wb three times (Corresponding to Fig 10c)

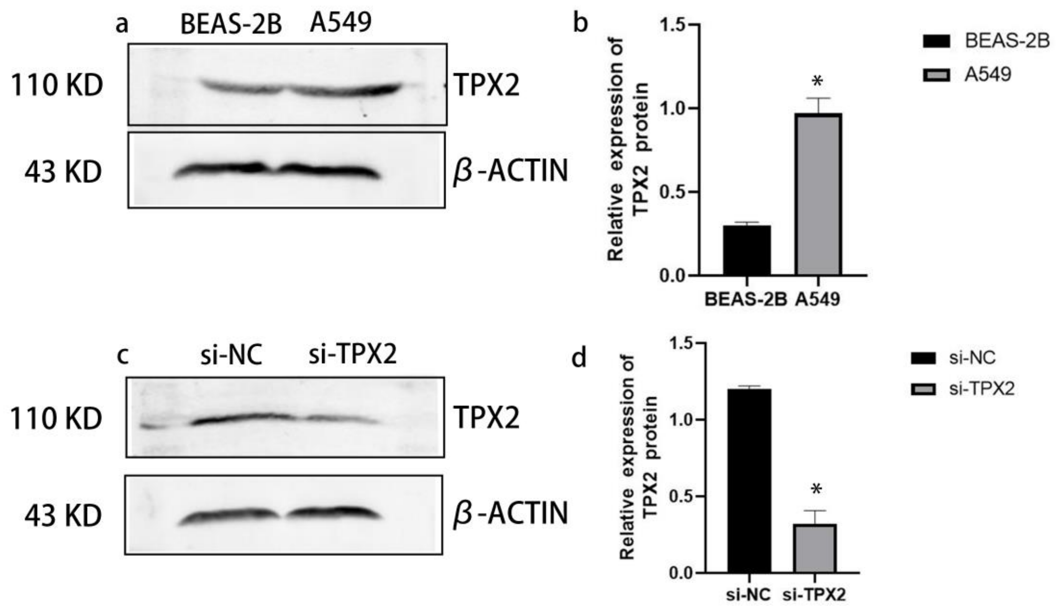

Fig. S3. Re-uploaded fig10.
